# Supplementary material for: Cell atlas of the developing human meninges reveals a dura origin of meningioma
Source: bioRxiv. 2025 Jul 13:2025.07.08.663122. Preprint. [Version 2] doi: 10.1101/2025.07.08.663122 (PMC12265625; doi:10.1101/2025.07.08.663122)
Supplement: Supplement 1 [file media-1.pdf]

Supplementary Materials for

Cell atlas of the developing human meninges reveals a dura origin of meningioma

Elin Vinsland[1,#], Sergio Marco Salas[2,3], Ivana Kapustová[1], Lijuan Hu[1], Simone Webb[4,5], Xiaofei Li[6], Xiaoling He[7], Mats Nilsson[2], Muzlifah Haniffa[5], Roger Barker[7], Oscar Persson[8,9], David R. Raleigh[10], Erik Sundström[6], Peter Lönnerberg[1], Sten Linnarsson[1,11,#]

#Corresponding authors: elin.vinsland@ki.se , sten.linnarsson@ki.se

The PDF file includes:

Figs. S1-S7

Table S1-S8 Legends

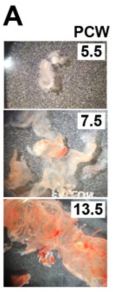

B **Samples**

10X run	Sample ID	Age, PCW	Tissue	Cells after QC	Mean genes	Mean UMIs	Sequencing saturation
297_5	XDD400	5.5	Meninges	13307	1987	6436	78.1%
256_1	BRC2243	6.6	Meninges	6352	2871	10555	84.4%
231_3, 231_4	BRC2218	6.7	Meninges	9894	2108	5973	79.3%, 80.3%
226_1	XHU364	7.5	Meninges	1646	2297	9355	80.5%
297_1, 297_2	XDD398	7.5	Meninges (Twins)	17328	2738	9737	50.4%, 56.3%
297_3, 297_4	XDD399	7.5	Meninges (Twins)	18946	3218	11115	46.3%, 41.3%
239_3, 239_4	BRC2227	7.6	Meninges	10433	2673	9039	70.9%, 68.4%
239_1, 239_2	BRC2226	8.4	Meninges	9550	3023	10724	58.4%, 58.7%
234_2, 234_3	BRC2222	9.0	Meninges	8848	2087	7001	70.5%, 71.6%
238_1, 238_2	BRC2225	9.9	Meninges	6180	2709	9392	72.9%, 59.0%
352_1	BRC2301	10.0	Meninges, FB dorsal	11190	2825	8713	86.7%
352_2	BRC2301	10.0	Meninges, FB ventral	11190	3048	8476	82.6%
352_3	BRC2301	10.0	Meninges, MB-HB dorsal	11691	2550	8782	89.7%
352_4	BRC2301	10.0	Meninges, MB-HB ventral	1246	897	2003	94.0%
184_1	XDD351	12.0	Meninges	2660	2389	10739	57.9%
256_2, 256_3	XDD385	13.5	Meninges	16265	3651	13433	69.3%, 70.3%

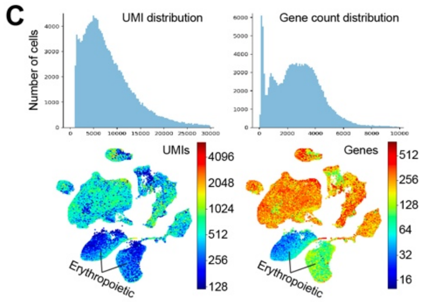

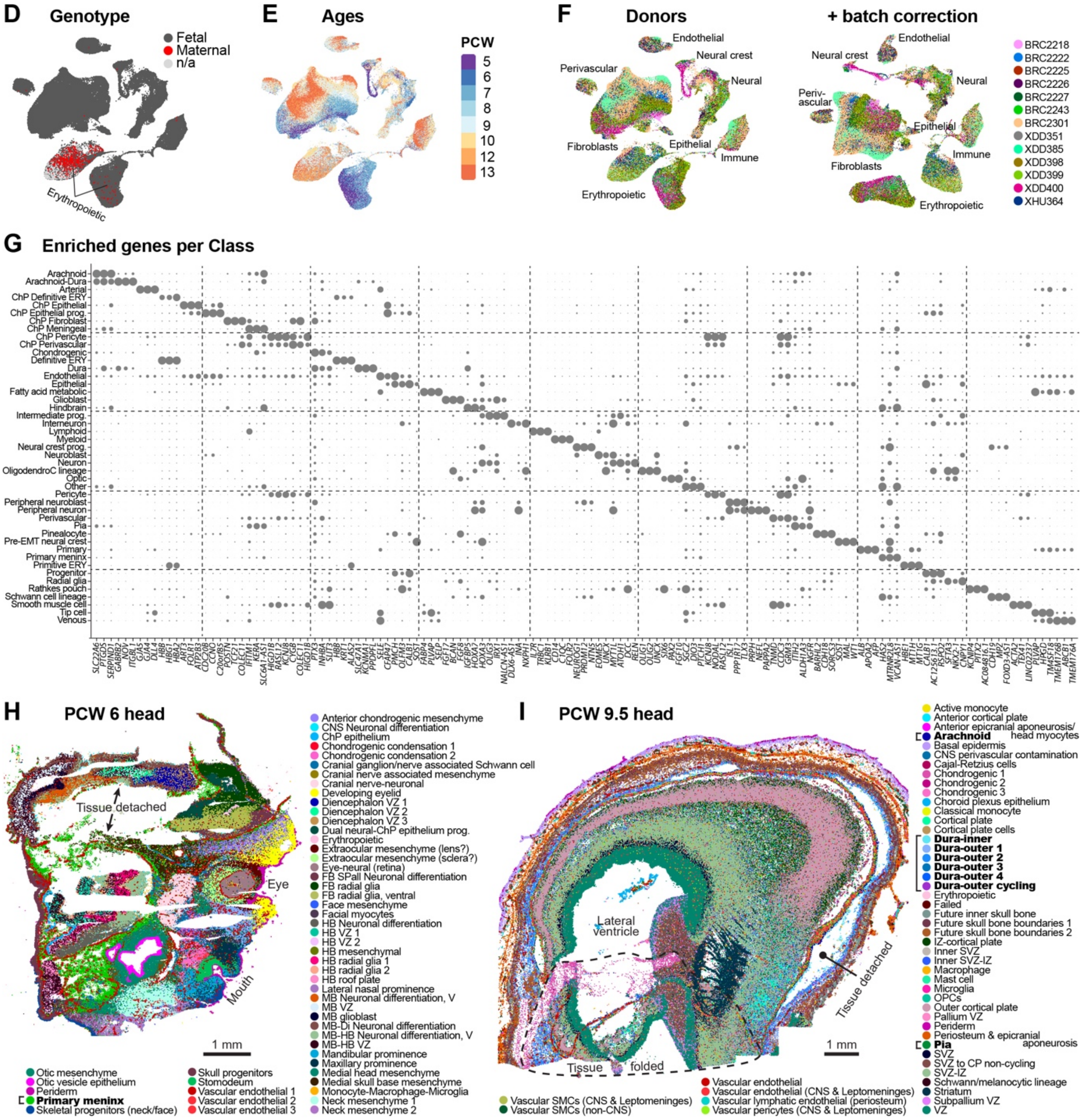

Figure S1. scRNA-seq QC and spatial clusters, related to Figure 1
(A) PCW 5.5, 7.5 and 13.5 meninges dissected off fetal brains.
(B) ScRNA-seq samples and sequencing information.
(C) Top: UMI distribution and gene count histograms. Bottom: UMAP embeddings of the scRNA-seq dataset, coloured by log UMIs and log genes per cell.
(D) UMAP coloured by maternal/fetal genotype, from Souporcell SNP analysis.
(E) UMAP coloured by sample ages.
(F) UMAPs without and with Harmony batch correction, coloured by donors.
(G) Dotplot of enriched genes per Class annotation. ChP, Choroid plexus; EMT, epithelial-mesenchymal transition; ERY, erythropoietic; OligodendroC, oligodendrocyte; prog., progenitor.
(H-I) Spatial transcriptomic clusters in sagittal sections of a PCW 6- (H) and PCW 9.5 (I) fetal head. Brackets and bold text highlight meninges-associated clusters. Dashed lines show where tissue folded before analysis. CP, cortical plate; Di, diencephalon; HB, hindbrain; IZ, intermediate zone; MB, midbrain; OPC, oligodendrocyte progenitor cell; SMC, smooth muscle cell; SVZ, subventricular zone; V, ventral; VZ, ventricular zone.

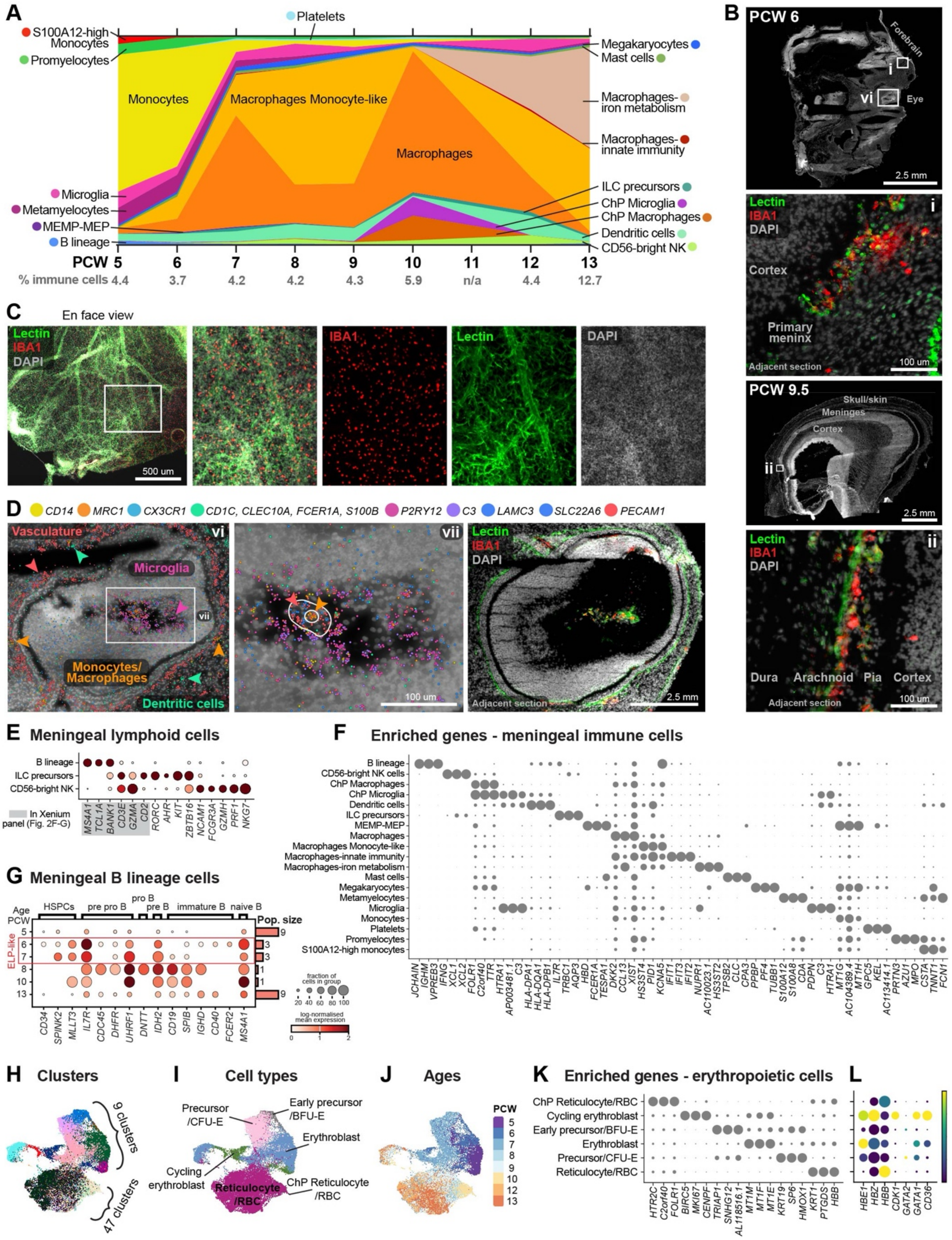
A
S100A12-high Monocytes
Promyelocytes
Platelets
Megakaryocytes
Mast cells
Monocytes
Macrophages Monocyte-like
Macrophages-iron metabolism
Macrophages-innate immunity
Macrophages
Microglia
Metamyelocytes
MEMP-MEP
B lineage
ILC precursors
ChP Microglia
ChP Macrophages
Dendritic cells
CD56-bright NK
PCW 5 6 7 8 9 10 11 12 13
% immune cells 4.4 3.7 4.2 4.2 4.3 5.9 n/a 4.4 12.7
B
PCW 6
Forebrain
Eye
2.5 mm
Lectin
IBA1
DAPI
Cortex
Primary meninx
Adjacent section
100 um
PCW 9.5
Skull/skin
Meninges
Cortex
Dura
Arachnoid
Pia
C
En face view
500 um
D
CD14
MRC1
CX3CR1
CD1C, CLEC10A, FCER1A, S100B
P2RY12
C3
LAMC3
SLC22A6
PECAM1
Vasculature
Microglia
Monocytes/ Macrophages
Dentritic cells
E Meningeal lymphoid cells
In Xenium panel (Fig. 2F-G)
F Enriched genes - meningeal immune cells
G Meningeal B lineage cells
HSPCs
pre pro B
pro B
pre B
immature B
naive B
Age PCW
ELP-like
Pop. size
fraction of cells in group
log-normalised mean expression
H Clusters
9 clusters
47 clusters
I Cell types
Precursor /CFU-E
Early precursor /BFU-E
Erythroblast
Cycling erythroblast
Reticulocyte /RBC
ChP Reticulocyte /RBC
J Ages
K Enriched genes - erythropoietic cells
L

Figure S2. Macrophages/microglia, lymphoid, and erythropoietic cells, related to Figure 2
(A) Proportion of immune cell types by age. In grey: proportion of immune cells by age compared to all cells in the meninges.
(B) IBA1 Immunostaining (macrophages and microglia), Lectin dye (vasculature), and DAPI (nuclei) in sagittal sections adjacent to sections used for spatial transcriptomics.
(C) As (B) but PCW 9.5 floating meninges.
(D) PCW 6 eye, with spatial transcriptomic data showing dots representing RNA molecules of markers for monocytes/macrophages, microglia, and dendritic cells, and IBA1 immunostaining.
(E) Dot plot showing the expression of lymphoid markers in a subset of meningeal lymphoid cells. Grey box indicates genes also included in the spatial transcriptomics (Xenium *in situ*) probe panel.
(F) Enriched genes for fetal meningeal immune cells.
(G) Dot plot of scRNAseq gene expression of B-lineage markers in developmental meninges across time (from 5-13 PCW). The size of the dot represents the % of cells within a group that express a given gene, and the colour of the dot indicates ln(x+1) mean gene expression within the group, where x equal counts normalised to 1e4 per cell. ELP, early lymphoid cell.
(H) UMAP of erythropoietic cells coloured by clusters.
(I) UMAP of erythropoietic cells coloured by cell type annotation.
(J) UMAP of erythropoietic cells coloured by sample ages.
(K) Enriched genes per erythropoietic cell annotation.
(L) Dot plot showing expression of yolk sac-derived primitive (*HBE1/HBZ*) and definitive (*HBB*) markers, and classical genes involved in erythropoiesis.

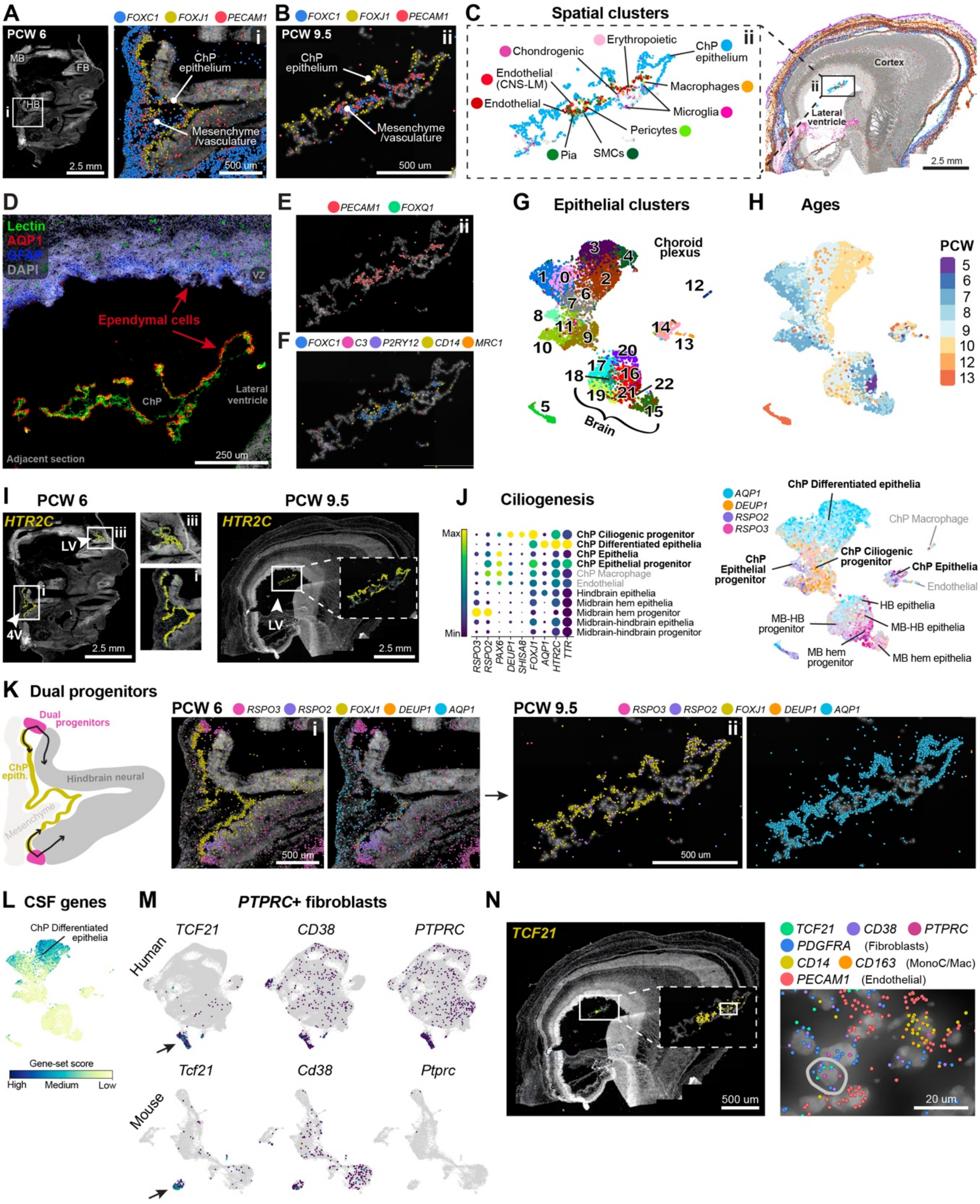
A
PCW 6
FOXC1 FOXJ1 PECAM1
MB
FB
HB
ChP epithelium
Mesenchyme /vasculature
2.5 mm
500 um
B
PCW 9.5
C
Spatial clusters
Chondrogenic
Erythropoietic
ChP epithelium
Endothelial (CNS-LM)
Endothelial
Macrophages
Microglia
Pericytes
Pia
SMCs
Cortex
Lateral ventricle
D
Lectin
AQP1
GFAP
DAPI
VZ
Ependymal cells
ChP
Lateral ventricle
Adjacent section
250 um
E
PECAM1 FOXQ1
F
FOXC1 C3 P2RY12 CD14 MRC1
G
Epithelial clusters
Choroid plexus
Brain
H
Ages
PCW
5
6
7
8
9
10
12
13
I
PCW 6
HTR2C
LV
4V
PCW 9.5
J
Ciliogenesis
Max
Min
ChP Ciliogenic progenitor
ChP Differentiated epithelia
ChP Epithelia
ChP Epithelial progenitor
ChP Macrophage
Endothelial
Hindbrain epithelia
Midbrain hem epithelia
Midbrain hem progenitor
Midbrain-hindbrain epithelia
Midbrain-hindbrain progenitor
RSPO3 RSPO2 PAX6 DEUP1 SHISA8 FOXJ1 AQP1 HTR2C TTR
AQP1
DEUP1
RSPO2
RSPO3
ChP Differentiated epithelia
ChP Macrophage
ChP Epithelial progenitor
ChP Ciliogenic progenitor
ChP Epithelia
Endothelial
HB epithelia
MB-HB progenitor
MB-HB epithelia
MB hem progenitor
MB hem epithelia
K
Dual progenitors
Dual progenitors
ChP epith.
Hindbrain neural
Mesenchyme
PCW 6
RSPO3 RSPO2 FOXJ1 DEUP1 AQP1
PCW 9.5
L
CSF genes
ChP Differentiated epithelia
Gene-set score
High
Medium
Low
M
PTPRC+ fibroblasts
Human
TCF21
CD38
PTPRC
Mouse
Tcf21
Cd38
Ptprc
N
TCF21
TCF21 CD38 PTPRC
PDGFRA (Fibroblasts)
CD14 CD163 (MonoC/Mac)
PECAM1 (Endothelial)
500 um
20 um

Figure S3. Fetal choroid plexus development, related to Figure 3
(A) Spatial transcriptomic data showing *FOXC1*+ mesenchymal/meningeal progenitors, endothelial cells (*PECAM1*), and choroid plexus (ChP) ependymal cells (*FOXJ1*) at PCW 6. FB, forebrain; MB, midbrain; HB, hindbrain.
(B) Same as (A) but at PCW 9.5.
(C) Annotated spatial clusters of the lateral ventricle ChP at PCW 9.5.
(D) Lectin dye (vasculature), DAPI (nuclei) and Aquaporin 1 (AQP1) immunostaining.
(E) Spatial transcriptomic data showing *PECAM1* and *FOXQ1* RNA molecules in the ChP stroma.
(F) Same as (E) but *CD14*/*MRC1*+ monocytes/macrophages, *FOXC1*+ ChP meningeal stroma, and *C3/P2RY12*+ microglia.
(G) scRNA-seq UMAP of ChP and brain epithelial cells, coloured by clusters.
(H) UMAP of epithelial cells coloured by sample ages.
(I) Spatial transcriptomics showing *HTR2C* expression.
(J) Dot plot showing the expression of genes involved in epithelial ciliogenesis (left), per annotated cell type on UMAP (right).
(K) Schematic and spatial validation of *RSPO2/3*+ dual neuro-epithelial progenitors, and ciliogenesis markers, at PCW 6 and 9.5.
(L) UMAP of epithelial cells, coloured by a CSF gene-set score (Methods).
(M) UMAPs of human fetal (this study) and mouse embryonic[41] meningeal fibroblasts coloured by expression of *TCF21/Tcf21*, *PTPRC/Ptprc*, and *CD38/Cd38* on a grey background of all cells.
(N) Spatial validation of *TCF21*+ *PTPRC*+ *CD38*+ fibroblasts in the ChP.

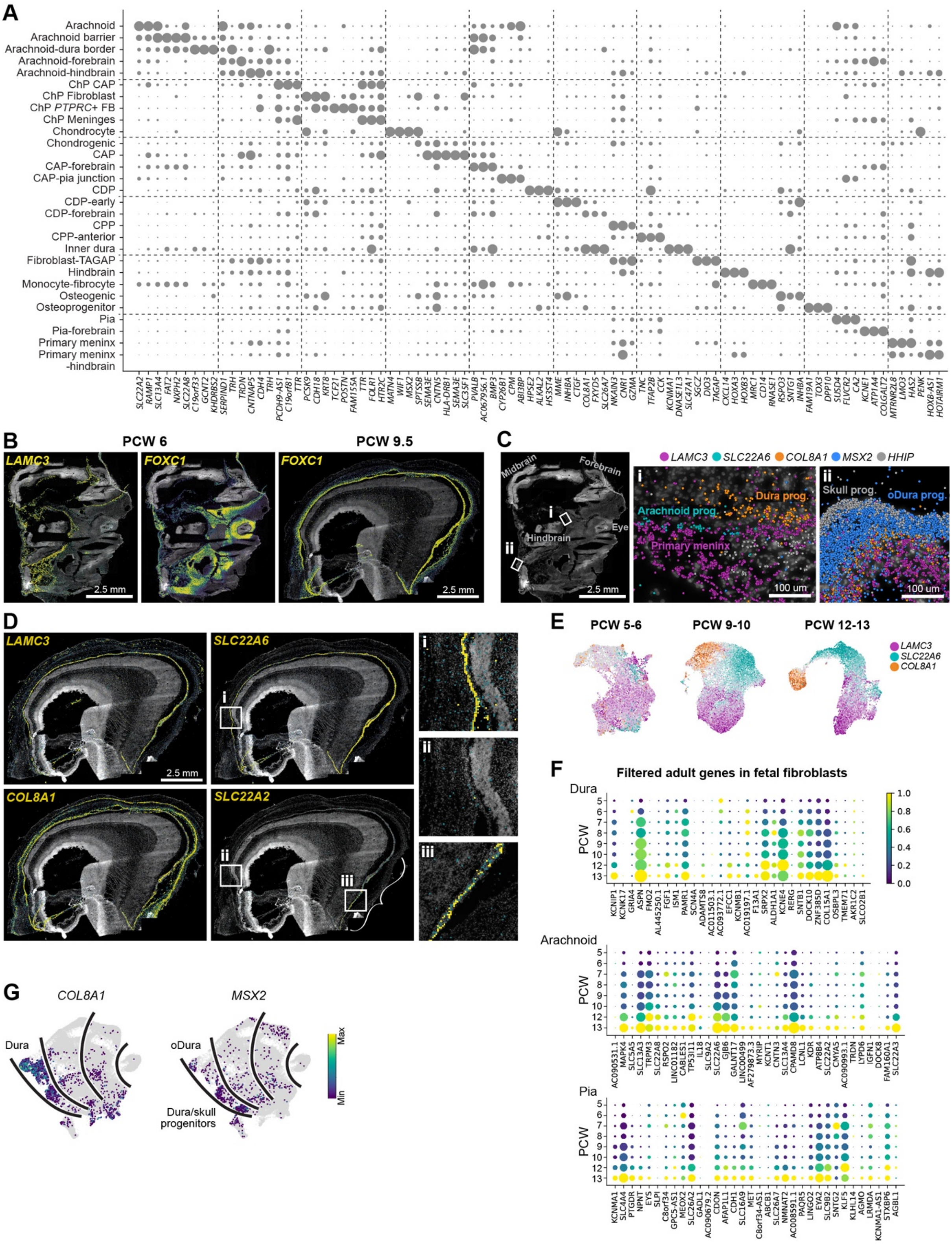

A
Arachnoid
Arachnoid barrier
Arachnoid-dura border
Arachnoid-forebrain
Arachnoid-hindbrain
ChP CAP
ChP Fibroblast
ChP PTPRC+ FB
ChP Meninges
Chondrocyte
Chondrogenic
CAP
CAP-forebrain
CAP-pia junction
CDP
CDP-early
CDP-forebrain
CPP
CPP-anterior
Inner dura
Fibroblast-TAGAP
Hindbrain
Monocyte-fibrocyte
Osteogenic
Osteoprogenitor
Pia
Pia-forebrain
Primary meninx
Primary meninx -hindbrain
B
PCW 6
PCW 9.5
LAMC3
FOXC1
FOXC1
2.5 mm
C
Midbrain
Forebrain
Hindbrain
Eye
LAMC3 SLC22A6 COL8A1 MSX2 HHIP
Dura prog.
Arachnoid prog.
Primary meninx
Skull prog.
oDura prog.
100 um
D
LAMC3
SLC22A6
COL8A1
SLC22A2
2.5 mm
E
PCW 5-6
PCW 9-10
PCW 12-13
LAMC3
SLC22A6
COL8A1
F
Filtered adult genes in fetal fibroblasts
Dura
Arachnoid
Pia
PCW
G
COL8A1
MSX2
Dura
oDura
Dura/skull progenitors
Max
Min

Figure S4. Meningeal layer marker dynamics, related to Figure 4
(A) Dotplot of enriched genes per CellType annotation. CAP, Committed arachnoid precursor; CDP, Committed dura precursor; ChP, Choroid plexus; CPP, Committed pia precursor.
(B) Spatial transcriptomics showing *LAMC3* and *FOXC1* expression at PCW 6, and *FOXC1* at 9.5.
(C) Spatial transcriptomics of identified layer markers *LAMC3* (pia), *SLC22A6* (arachnoid), *COL8A1* (dura), *MSX2* (outer dura, skull and periosteum), and *HHIP* (skull) at PCW 6.
(D) Spatial transcriptomics showing *LAMC3, SLC22A6, COL8A1,* and *SLC22A2* expression at PCW 9.5.
(E) UMAPs of subsets of pooled PCW 5-6, 9-10, and 12-13 fibroblasts. Cells are coloured by their expression of the gene they express most highly among *LAMC3* (pia), *SLC22A6* (arachnoid), *COL8A1* (dura).
(F) Dotplot showing the expression of enriched genes from adult pia, arachnoid, and dura (Fig. 4F), in fetal fibroblasts over developmental timepoints.
(G) UMAP showing the expression of *COL8A1* (dura) and *MSX2* (outer dura) in fetal meningeal fibroblasts.

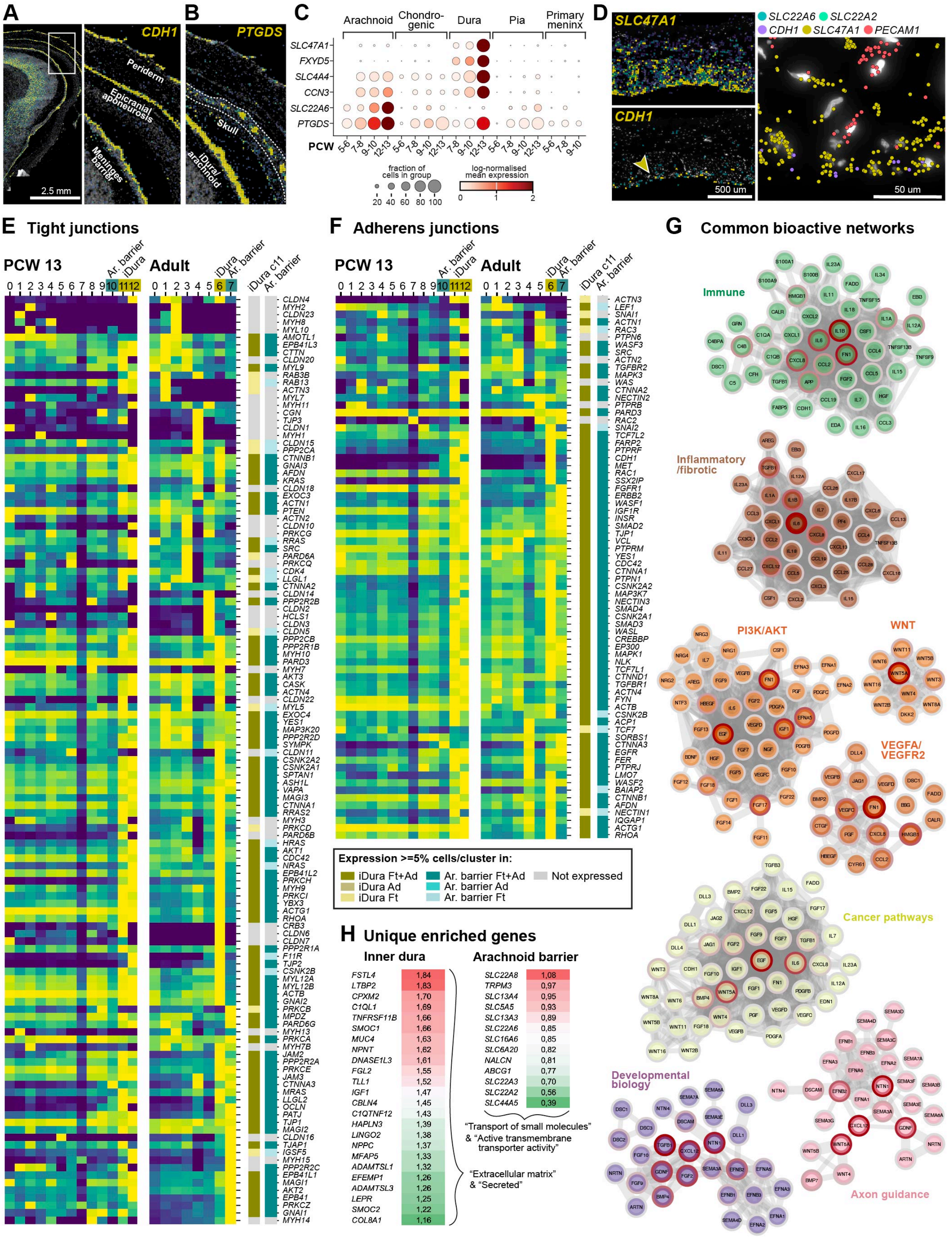
A
CDH1
Periderm
Epicranial aponeurosis
Meninges barrier
2.5 mm
B
PTGDS
Skull
iDura/arachnoid
C
Arachnoid
Chondro-genic
Dura
Pia
Primary meninx
SLC47A1
FXYD5
SLC4A4
CCN3
SLC22A6
PTGDS
PCW
5-6
7-8
9-10
12-13
fraction of cells in group
20 40 60 80 100
log-normalised mean expression
0 1 2
D
SLC47A1
CDH1
SLC22A6
SLC22A2
SLC47A1
PECAM1
500 um
50 um
E Tight junctions
PCW 13
Adult
Ar. barrier
iDura
iDura c11
Ar. barrier
0 1 2 3 4 5 6 7 8 9 10 11 12
0 1 2 3 4 5 6 7
CLDN4 MYH2 CLDN23 MYH8 MYL10 AMOTL1 EPB41L3 CTTN CLDN20 MYL9 RAB3B RAB13 ACTN3 MYL7 MYH11 CGN TJP3 CLDN1 MYH1 CLDN15 PPP2CA CTNNB1 GNAI3 AFDN KRAS CLDN18 EXOC3 ACTN1 PTEN ACTN2 CLDN10 PRKCG RRAS SRC PARD6A PRKCQ CDK4 LLGL1 CTNNA2 CLDN14 PPP2R2B CLDN2 HCLS1 CLDN3 CLDN5 PPP2CB PPP2R1B MYH10 PARD3 MYH7 AKT3 CASK ACTN4 CLDN22 MYL5 EXOC4 YES1 MAP3K20 PPP2R2D SYMPK CLDN11 CSNK2A2 CSNK2A1 SPTAN1 ASH1L VAPA MAGI3 CTNNA1 RRAS2 MYH3 PRKCD PARD6B HRAS AKT1 CDC42 NRAS EPB41L2 PRKCH MYH9 PRKCI YBX3 ACTG1 RHOA CRB3 CLDN6 CLDN7 PPP2R1A F11R TJP2 CSNK2B MYL12A MYL12B ACTB GNAI2 PRKCB MPDZ PARD6G MYH13 PRKCA MYH7B JAM2 PPP2R2A PRKCE JAM3 CTNNA3 MRAS LLGL2 OCLN PATJ TJP1 MAGI2 CLDN16 TJAP1 IGSF5 MYH15 PPP2R2C EPB41L1 MAGI1 AKT2 EPB41 PRKCZ GNAI1 MYH14
F Adherens junctions
PCW 13
Adult
ACTN3 LEF1 SNAI1 ACTN1 RAC3 PTPN6 WASF3 SRC TGFBR2 MAPK3 WAS CTNNA2 NECTIN2 PTPRB PARD3 RAC2 SNAI2 TCF7L2 FARP2 PTPRF CDH1 MET RAC1 SSX2IP FGFR1 ERBB2 WASF1 IGF1R INSR SMAD2 TJP1 VCL PTPRM YES1 CDC42 CTNNA1 PTPN1 CSNK2A2 MAP3K7 NECTIN3 SMAD4 CSNK2A1 SMAD3 WASL CREBBP EP300 MAPK1 NLK TCF7L1 CTNND1 TGFBR1 ACTN4 FYN ACTB CSNK2B ACP1 TCF7 SORBS1 CTNNA3 EGFR FER PTPRJ LMO7 WASF2 BAIAP2 CTNNB1 AFDN NECTIN1 IQGAP1 ACTG1 RHOA
Expression >=5% cells/cluster in:
iDura Ft+Ad
iDura Ad
iDura Ft
Ar. barrier Ft+Ad
Ar. barrier Ad
Ar. barrier Ft
Not expressed
H Unique enriched genes
Inner dura
FSTL4 1,84
LTBP2 1,83
CPXM2 1,70
C1QL1 1,69
TNFRSF11B 1,66
SMOC1 1,66
MUC4 1,63
NPNT 1,62
DNASE1L3 1,61
FGL2 1,55
TLL1 1,52
IGF1 1,47
CBLN4 1,45
C1QTNF12 1,43
HAPLN3 1,39
LINGO2 1,38
NPPC 1,37
MFAP5 1,33
ADAMTSL1 1,32
EFEMP1 1,26
ADAMTSL3 1,26
LEPR 1,25
SMOC2 1,22
COL8A1 1,16
Arachnoid barrier
SLC22A8 1,08
TRPM3 0,97
SLC13A4 0,95
SLC5A5 0,93
SLC13A3 0,89
SLC22A6 0,85
SLC16A6 0,85
SLC6A20 0,82
NALCN 0,81
ABCG1 0,77
SLC22A3 0,70
SLC22A2 0,56
SLC44A5 0,39
"Transport of small molecules" & "Active transmembrane transporter activity"
"Extracellular matrix" & "Secreted"
G Common bioactive networks
Immune
Inflammatory /fibrotic
PI3K/AKT
WNT
VEGFA/VEGFR2
Cancer pathways
Developmental biology
Axon guidance

Figure S5. Shared and unique gene expression in inner dura and arachnoid barrier cells, related to Figure 5

(A) Spatial transcriptomics showing *CDH1* expression in PCW 9.5 head fibroblasts.
(B) Same as (A) but *PTGDS* expression.
(C) Dotplot showing the expression of arachnoid and dura-specific genes over time, in arachnoid, chondrogenic, dura, pia, and primary meninx.
(D) Spatial transcriptomics showing *SLC47A1*, *CDH1*, *SLC22A2*, *SLC22A6* and *PECAM1* expression in human adult dura.
(E) Heatmap showing the expression of genes from the full KEGG list of tight junction genes (#M11355), in scRNA-seq clusters of PCW 13 and adult meningeal fibroblast. Right: Stylised heatmaps with a threshold criteria of genes being expressed in at least 5% of cells/cluster.
(F) Same as (E) but the full KEGG list of adherens junction genes (#M638).
(G) STRING interaction networks of shared bioactive genes between inner dura and arachnoid barrier clusters, split into subsets of significantly enriched functional pathways. Gene centrality (connectedness) outlined by red.
(H) Subset of enriched genes in inner dura and arachnoid barrier clusters, and relevant ontologies.

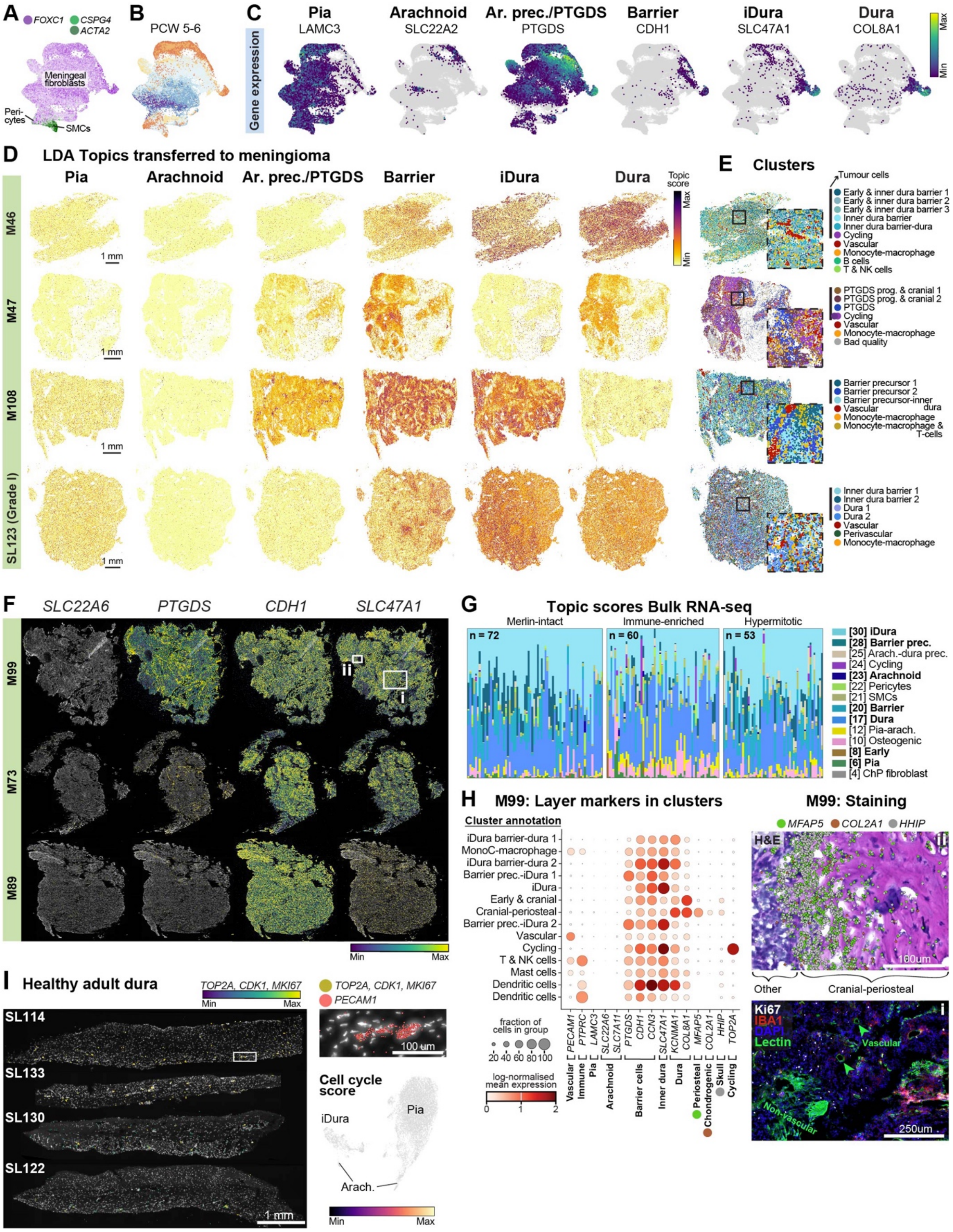
A
FOXC1
CSPG4
ACTA2
Meningeal fibroblasts
Peri-cytes
SMCs
B
PCW 5-6
C
Gene expression
Pia
LAMC3
Arachnoid
SLC22A2
Ar. prec./PTGDS
PTGDS
Barrier
CDH1
iDura
SLC47A1
Dura
COL8A1
Max
Min
D
LDA Topics transferred to meningioma
Pia
Arachnoid
Ar. prec./PTGDS
Barrier
iDura
Dura
Topic score
Max
Min
M46
M47
M108
SL123 (Grade I)
1 mm
E
Clusters
Tumour cells
Early & inner dura barrier 1
Early & inner dura barrier 2
Early & inner dura barrier 3
Inner dura barrier
Inner dura barrier-dura
Cycling
Vascular
Monocyte-macrophage
B cells
T & NK cells
PTGDS prog. & cranial 1
PTGDS prog. & cranial 2
PTGDS
Cycling
Vascular
Monocyte-macrophage
Bad quality
Barrier precursor 1
Barrier precursor 2
Barrier precursor-inner dura
Vascular
Monocyte-macrophage
Monocyte-macrophage & T-cells
Inner dura barrier 1
Inner dura barrier 2
Dura 1
Dura 2
Vascular
Perivascular
Monocyte-macrophage
F
SLC22A6
PTGDS
CDH1
SLC47A1
M99
M73
M89
ii
i
Min
Max
G
Topic scores Bulk RNA-seq
Merlin-intact
Immune-enriched
Hypermitotic
n = 72
n = 60
n = 53
[30] iDura
[28] Barrier prec.
[25] Arach.-dura prec.
[24] Cycling
[23] Arachnoid
[22] Pericytes
[21] SMCs
[20] Barrier
[17] Dura
[12] Pia-arach.
[10] Osteogenic
[8] Early
[6] Pia
[4] ChP fibroblast
H
M99: Layer markers in clusters
Cluster annotation
iDura barrier-dura 1
MonoC-macrophage
iDura barrier-dura 2
Barrier prec.-iDura 1
iDura
Early & cranial
Cranial-periosteal
Barrier prec.-iDura 2
Vascular
Cycling
T & NK cells
Mast cells
Dendritic cells
Dendritic cells
fraction of cells in group
20 40 60 80 100
log-normalised mean expression
0 1 2
PECAM1
PTPRC
LAMC3
SLC22A6
SLC7A11
PTGDS
CDH1
CCN3
SLC47A1
KCNMA1
COL8A1
MFAP5
COL2A1
HHIP
TOP2A
Vascular
Immune
Pia
Arachnoid
Barrier cells
Inner dura
Dura
Periosteal
Chondrogenic
Skull
Cycling
M99: Staining
MFAP5
COL2A1
HHIP
H&E
ii
100um
Other
Cranial-periosteal
Ki67
IBA1
DAPI
Lectin
i
Vascular
Non-vascular
250um
I
Healthy adult dura
TOP2A, CDK1, MKI67
Min
Max
TOP2A, CDK1, MKI67
PECAM1
SL114
SL133
SL130
SL122
1 mm
100 um
Cell cycle score
iDura
Pia
Arach.
Min
Max

Figure S6. Further analysis demonstrating that meningioma tumours are dura-like, related to Figure 6

(A) Cells coloured by their expression of the gene they express most highly among *FOXC1* (meningeal fibroblasts & pericytes), *CSPG4* (pericytes), *ACTA2* (SMCs).
(B) UMAP of fetal fibroblasts and perivascular cells coloured by sample ages.
(C) Genes ranked highly by our LDA model for meningeal layer topics. Gene expression shown on UMAP of fetal fibroblasts and perivascular cells, on grey background of all cells. Ar. prec., Arachnoid precursors; iDura, inner dura.
(D) LDA topics transferred to spatial data from three high grade hypermitotic meningioma tumours; M46, M47 & M108, and one grade I tumour; SL123.
(E) Spatial clusters of tumours shown in (D), coloured by their most similar meningeal layer.
(F) Spatial transcriptomic data showing *SLC22A6*, *PTGDS*, *CDH1*, and *SLC47A1* expression in M99, M73, and M89 tumours. Inset references i and ii are for (H).
(G) Stacked bar chart showing scores of LDA topics transferred to bulk RNA-sequencing data from 185 meningioma tumours[18].
(H) Left: Dot plot showing gene expression of meningeal layer markers in M99 clusters. MonoC, Monocytes. Top right: Spatial transcriptomic data of *MFAP5*, *COL2A1* and *HHIP*, on H&E staining. Bottom right: Immunohistochemistry of Ki67, and IBA1, and DAPI and Lectin dyes. Inset references i and ii can be seen in (F).
(I) Spatial transcriptomic data of four human adult dura samples, showing RNA molecules of cycling cells (*TOP1A, CDK1, MKI67*) and endothelial cells (*PECAM1*). Cell cycle score on scRNA-seq UMAP of adult fibroblasts.

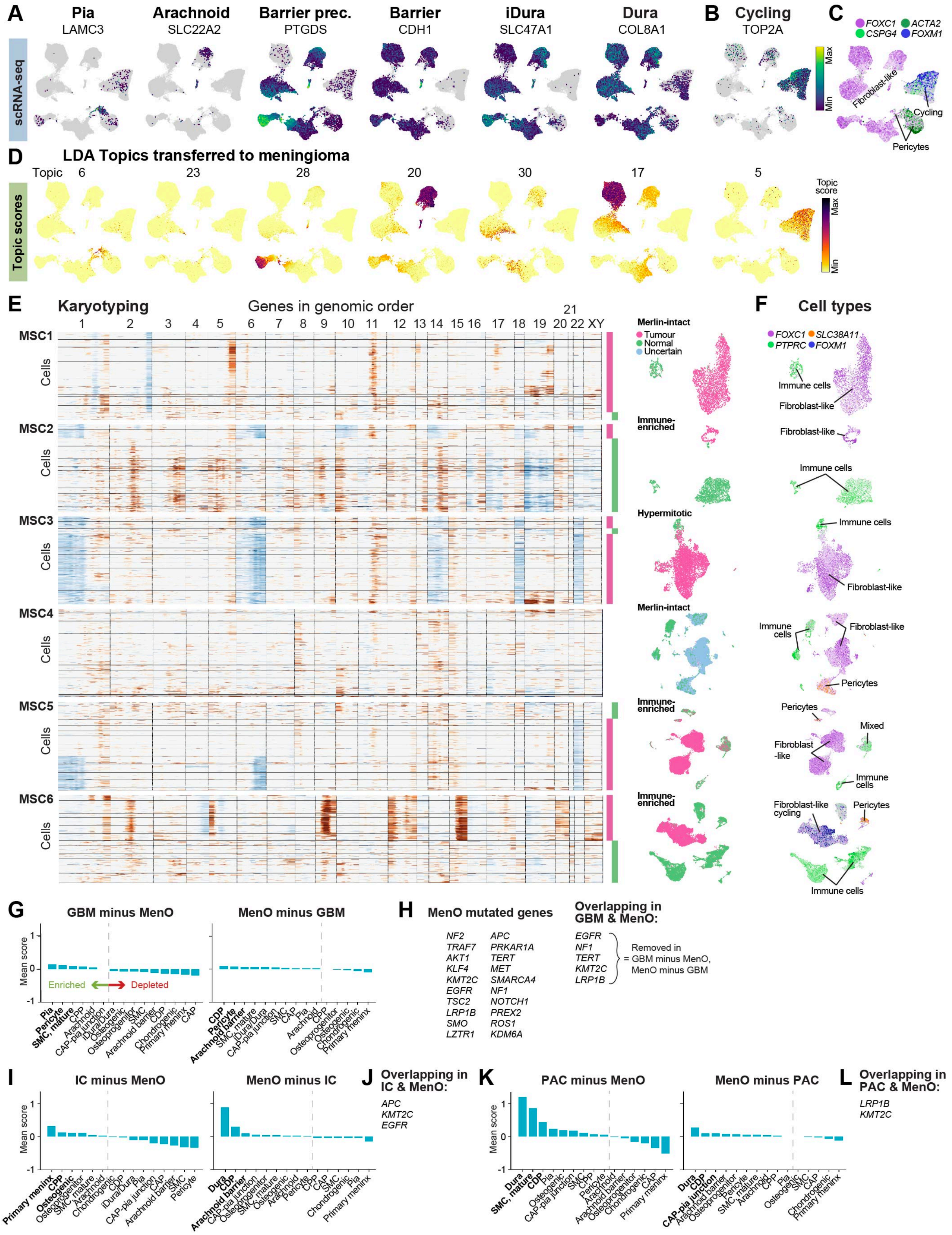

A
Pia
LAMC3
Arachnoid
SLC22A2
Barrier prec.
PTGDS
Barrier
CDH1
iDura
SLC47A1
Dura
COL8A1
B
Cycling
TOP2A
C
FOXC1
ACTA2
CSPG4
FOXM1
scRNA-seq
Max
Min
Fibroblast-like
Cycling
Pericytes
D
LDA Topics transferred to meningioma
Topic 6
23
28
20
30
17
5
Topic scores
Topic score
Max
Min
E
Karyotyping
Genes in genomic order
1 2 3 4 5 6 7 8 9 10 11 12 13 14 15 16 17 18 19 20 21 22 XY
MSC1
MSC2
MSC3
MSC4
MSC5
MSC6
Cells
Merlin-intact
Tumour
Normal
Uncertain
Immune-enriched
Hypermitotic
Merlin-intact
Immune-enriched
Immune-enriched
F
Cell types
FOXC1
SLC38A11
PTPRC
FOXM1
Immune cells
Fibroblast-like
Fibroblast-like
Immune cells
Immune cells
Fibroblast-like
Immune cells
Fibroblast-like
Pericytes
Pericytes
Mixed
Fibroblast -like
Immune cells
Fibroblast-like cycling
Pericytes
Immune cells
G
GBM minus MenO
MenO minus GBM
Mean score
Enriched
Depleted
Pia
Pericyte
SMC, mature
CPP
Arachnoid
CAP-pia junction
iDura/Dura
Osteogenic
Osteoprogenitor
SMC
Arachnoid barrier
CDP
Chondrogenic
Primary meninx
CAP
H
MenO mutated genes
NF2
TRAF7
AKT1
KLF4
KMT2C
EGFR
TSC2
LRP1B
SMO
LZTR1
APC
PRKAR1A
TERT
SMARCA4
MET
NF1
NOTCH1
PREX2
ROS1
KDM6A
Overlapping in GBM & MenO:
EGFR
NF1
TERT
KMT2C
LRP1B
= Removed in GBM minus MenO, MenO minus GBM
I
IC minus MenO
MenO minus IC
Mean score
J
Overlapping in IC & MenO:
APC
KMT2C
EGFR
K
PAC minus MenO
MenO minus PAC
Mean score
L
Overlapping in PAC & MenO:
LRP1B
KMT2C

Figure S7. Analysis of meningioma scRNA-seq, and mutated genes from various cancers in fetal meningeal fibroblasts, related to Figure 7

(A) UMAP of tumour cells, coloured by the expression of genes ranked highly by our LDA model for meningeal layer topics, on a grey background of all cells.
(B) UMAP of tumour cells, coloured by *TOP2A* expression.
(C) Cells coloured by their expression of the gene they express most highly among *FOXC1* (meningeal fibroblasts & pericytes), *CSPG4* (pericytes), *ACTA2* (SMCs), and *FOXM1* (cycling tumour).
(D) LDA topics for meningeal layers and cycling cells (Topic 5) transferred to meningioma scRNA-seq data.
(E) Karyotyping of individual tumours. Chromosomal deletions are blue, and gains are brown. Bars to the right and UMAPs of individual tumours indicate cells that are tumour (pink), normal (green) and of uncertain karyotype (light blue).
(F) Annotation of individual tumour samples, and expression of *FOXC1* (meningeal), *SLC38A11* (pericytes), *ACTA2* (SMCs), *PTPRC* (immune).
(G) Quantification of gene-set score of top 20 mutated genes in glioblastoma (GBM) tumours minus genes overlapping with top 20 mutated genes in meningioma (MenO), and MenO minus GBM overlapping genes, in fetal meningeal fibroblast and perivascular cell Subclass (as in Figure 7F).
(H) Top 20 mutated genes in meningioma, and genes overlapping between the top 20 mutated genes in GBM and MenO.
(I) Same as (G) but for intestinal carcinomas (IC).
(J) List of overlapping genes between the top 20 mutated genes in IC and MenO.
(K) Same as (G) but for prostate adenocarcinomas (PAC).
(L) List of overlapping genes between the top 20 mutated genes in PAC and MenO.

Table S1. Samples

Table S2. scRNA-seq cluster metadata

Table S3. Xenium probe panels

Table S4. Xenium clusters

Table S5. CSF analysis, related to Figure S3

Table S6. Network analysis, related to Figures 5 and S5

Table S7. Topics filtered genes, related to Figures 6, S6, and S7

Table S8. COSMIC mutated genes, related to Figure 7 and S7
